# Letter to the Editor—Comment on “Erythropoietic Protoporphyria and Surgery: A Case of Successful Mastectomy With Perioperative Light Shielding Strategies Based on Light‐Induced Hemolytic Threshold Assessment”

**DOI:** 10.1111/1346-8138.70386

**Published:** 2026-07-22

**Authors:** Ingeborg de Rover, N. Chantal Peltenburg, Johan C. M. Sweep, Janneke G. Langendonk

**Affiliations:** ^1^ Department of Internal Medicine, Porphyria Expert Center Rotterdam, Center for Lysosomal and Metabolic Diseases Erasmus MC Rotterdam the Netherlands; ^2^ Department of Information and Technology Erasmus MC Rotterdam the Netherlands

1

We read with great interest the report by Tateishi et al. [1] on perioperative light shielding to prevent light‐induced complications in erythropoietic protoporphyria (EPP). EPP is characterized by accumulation of protoporphyrin IX (PPIX), leading to phototoxic injury upon exposure to visible (blue) light [2]. This can result in endothelial damage and, after prolonged exposure, pain, edema, and tissue necrosis [3].

Perioperative management in EPP is focused on preventing pain and tissue necrosis. It should be guided by structured patient‐specific risk stratification incorporating hepatic function and PPIX levels [4]. Three domains are critical: (1) prevention of cutaneous and visceral phototoxic injury; (2) avoidance of hepatotoxic agents and iron supplementation, which may precipitate cholestatic hepatitis; and (3) selective use of adjunctive measures, such as preoperative PPIX reduction. Although intraoperative phototoxicity risk is generally low, it increases with hepatic dysfunction, prolonged open procedures (> 3 h), high‐intensity lighting, and elevated PPIX concentrations (> 60 μmol/L; ~3378 μg/dL). Mitigation relies on minimizing light exposure and shielding vulnerable tissues, which is typically sufficient in minimally invasive procedures. In higher‐risk settings, additional measures such as blue‐light filtering and preoperative exchange transfusion may be required. Iron supplementation and hepatotoxic medications should be avoided to prevent further PPIX accumulation and hepatic injury.

We report three cases illustrating different perioperative management strategies, including locally developed optical filters in which a film was affixed to a circular plexiglass plate [5]. This reduced overall operating‐room light intensity from 9000 to 3000 lx and decreased the proportion of blue and ultraviolet light below 500 nm from 73% to 35%. A 31‐year‐old female with congenital erythropoietic protoporphyria (CEP), another porphyria with severe cutaneous photosensitivity (uroporphyrin/creatinine 1856 nmol/mmol, reference < 2.0 nmol/mmol; heptaporphyrin/creatinine 60 nmol/mmol, reference < 1.6 nmol/mmol) underwent elective caesarean section using light‐filtering measures. The surgery was uneventful, with no reported phototoxic or hepatic complications.

Another representative case involved a 53‐year‐old woman with EPP scheduled for mastectomy (duration of 4 h), followed later by a shorter reconstructive procedure. Given extremely elevated PPIX levels (275 μmol/L; 15 483 μg/dL; reference < 1.5 μmol/L) with normal liver enzymes and function, preoperative erythrocyte exchange transfusions (two sessions, eight units) were performed, reducing PPIX to 53 μmol/L. In this case, surgery with filtered lighting was also uneventful.

The third patient, a 78 year‐old male, was scheduled for a Transcatheter Aortic Valve Implantation (TAVI) with potential conversion to thoracotomy. As the procedure occurred at an alternative, preferred hospital where light filters were unavailable, a preoperative erythrocyte exchange transfusion (one session with eight units) was planned. PPIX reduced from 72 to 31 μmol/L with substantially reduced postoperative EPP symptoms. Conversion to thoracotomy appeared unnecessary.

These cases illustrate the importance of individualized perioperative risk assessments in EPP, particularly given the limited availability and cost considerations of light filters and erythrocyte exchange transfusions. Light‐filtering measures should be prioritized, with reduction of circulating PPIX reserved for high‐risk settings. Safe perioperative care further requires avoidance of iron supplementation and hepatotoxic agents, alongside close monitoring of hepatic status and porphyrin burden.

## Ethics Statement

The authors have nothing to report. Informed Consent, Registry and the Registration and Animal studies: N/A.

## Conflicts of Interest

Dr. J.G. Langendonk receives an unrestricted grant from Clinuvel LTD, 2025–2030, paid to institute. She is/was also a principal investigator for industry sponsored phase I to IV trials, sponsored by Alnylam, Ultragenyx, Moderna, Clinuvel, and Disc medicine, also paid to institute.

## Data Availability

The data that support the findings of this study are available on request from the corresponding author. The data are not publicly available due to privacy or ethical restrictions.
